# Cell-free DNA as a potential alternative to genomic DNA in genetic studies

**DOI:** 10.1093/nargab/lqaf119

**Published:** 2025-09-09

**Authors:** Jingyu Zeng, Huanhuan Zhu, Yu Wang, Guodan Zeng, Panhong Liu, Rijing Ou, Xianmei Lan, Yuhui Zheng, Chenhui Zhao, Linxuan Li, Haiqiang Zhang, Jianhua Yin, Mingzhi Liao, Yan Zhang, Xin Jin

**Affiliations:** College of Life Sciences, Northwest A&F University, Yangling, Shaanxi 712100, China; BGI Research, Shenzhen 518083, China; Shenzhen Key Laboratory of Transomics Biotechnologies, BGI Research, Shenzhen 518083, China; BGI Research, Shenzhen 518083, China; Shenzhen Key Laboratory of Transomics Biotechnologies, BGI Research, Shenzhen 518083, China; State Key Laboratory of Genome and Multi-Omics Technologies, BGI Research, Shenzhen518083, China; BGI Research, Shenzhen 518083, China; Shenzhen Key Laboratory of Transomics Biotechnologies, BGI Research, Shenzhen 518083, China; BGI Research, Shenzhen 518083, China; College of Life Sciences, University of Chinese Academy of Sciences, Beijing 100049, China; BGI Research, Shenzhen 518083, China; Shenzhen Key Laboratory of Transomics Biotechnologies, BGI Research, Shenzhen 518083, China; BGI Research, Shenzhen 518083, China; BGI Research, Shenzhen 518083, China; Shenzhen Key Laboratory of Transomics Biotechnologies, BGI Research, Shenzhen 518083, China; College of Life Sciences, University of Chinese Academy of Sciences, Beijing 100049, China; BGI Research, Shenzhen 518083, China; BGI Research, Shenzhen 518083, China; BGI Research, Shenzhen 518083, China; Shenzhen Key Laboratory of Transomics Biotechnologies, BGI Research, Shenzhen 518083, China; College of Life Sciences, University of Chinese Academy of Sciences, Beijing 100049, China; BGI Research, Shenzhen 518083, China; Shenzhen Key Laboratory of Transomics Biotechnologies, BGI Research, Shenzhen 518083, China; BGI Research, Shenzhen 518083, China; State Key Laboratory of Genome and Multi-Omics Technologies, BGI Research, Shenzhen518083, China; College of Life Sciences, Northwest A&F University, Yangling, Shaanxi 712100, China; BGI Research, Shenzhen 518083, China; State Key Laboratory of Genome and Multi-Omics Technologies, BGI Research, Shenzhen518083, China; BGI Research, Shenzhen 518083, China; Shenzhen Key Laboratory of Transomics Biotechnologies, BGI Research, Shenzhen 518083, China; The Innovation Centre of Ministry of Education for Development and Diseases, School of Medicine, South China University of Technology, Guangzhou 510006, China; Shanxi Medical University-BGI Collaborative Center for Future Medicine, Shanxi Medical University, Taiyuan 030001, China; State Key Laboratory of Genome and Multi-Omics Technologies, BGI Research, Shenzhen518083, China

## Abstract

Next-generation sequencing has greatly advanced genomics, enabling large-scale studies of population genetics and complex traits. Genomic DNA (gDNA) from white blood cells has traditionally been the main data source, but cell-free DNA (cfDNA), found in bodily fluids as fragmented DNA, is increasingly recognized as a valuable biomarker in clinical and genetic studies. However, a direct comparison between cfDNA and gDNA has not been fully explored. In this study, we analyzed cfDNA and gDNA from 186 healthy individuals, using the same sequencing platform. We compared sequencing quality, variant detection, allele frequencies (AF), genotype concordance, population structure, and genomic association results (genome-wide association study and expression quantitative trait locus). While cfDNA showed higher duplication rates and lower effective sequencing depth, both DNA types displayed similar quality metrics at the same depth. We also observed that significant depth differences between cfDNA and gDNA were mainly found in centromeric regions. While gDNA identified more variants with more uniform coverage, AF spectra, population structure, and genomic associations were largely consistent between the two DNA types. This study provides a detailed comparison of cfDNA and gDNA, highlighting the potential of cfDNA as an alternative to gDNA in genomic research. Our findings could serve as a reference for future studies on cfDNA and gDNA.

## Introduction

In recent years, advancements in sequencing technology, particularly next-generation sequencing, have significantly accelerated research in genomics and genetics [1]. As a result, numerous large-scale genomic cohorts have been established to explore the genetic history of populations and the genetic underpinnings of complex diseases and traits. Notable examples include the 1KGP (1000 Genomes Project) [2], the gnomAD (Genome Aggregation Database) [3], the TOPMed (Trans-Omics for Precision Medicine) project [4], the UKB (UK Biobank) [5], the ChinaMAP (China Metabolic Analytics Project) [6], and the CKB (China Kadoorie Biobank) [7].

The whole-genome sequencing (WGS) data in these cohorts is typically derived from cellular genomic DNA (gDNA), which is extracted from the nuclei of white blood cells. Under normal conditions, gDNA consists of long, complete double-helix strands. During library preparation, the long DNA molecules are fragmented into pieces of a specific length and subsequently sequenced using sequencing platforms. gDNA data serves as a cornerstone in a variety of genomic and genetic research fields, including population genetics [8, 9], pharmacogenomics [10, 11], functional genomics [12], genome-wide association studies (GWAS) [13], polygenic risk score [14], and Mendelian randomization analysis [15].

Cell-free DNA (cfDNA) refers to fragmented DNA released into various body fluids such as blood plasma, urine, and cerebrospinal fluid [16]. The sources of cfDNA are diverse and depend on the physiological condition of the host. These sources include dying host cells, cell-free fetal DNA (cffDNA), circulating tumor DNA, circulating microbial DNA, mitochondrial DNA, and transplanted organs. Initially thought to be cellular waste, cfDNA has since been recognized as a valuable biomarker that reflects the physiological state of the body. For example, cffDNA is released into maternal plasma via the placenta. By drawing blood from pregnant women and sequencing plasma DNA using WGS technology, chromosomal disorders in the fetus (e.g. Down syndrome) can be detected. This is the basis of the well-known noninvasive prenatal testing technology [17]. cfDNA testing is also used for the early detection of certain cancers [18]. Differences in molecular characteristics, such as fragment size, between tumor-derived cfDNA and host-derived cfDNA can serve as potential biomarkers for cancer diagnosis and monitoring.

In recent years, cfDNA applications have expanded from clinical testing to population genetics and GWAS. The investigated traits encompass a wide range of maternal and neonatal measurements, including prenatal tests, maternal and neonatal metabolites, and pregnancy complications [19–23]. Additionally, research has expanded beyond trait associations to explore cfDNA molecular features, such as concentration and end motif frequencies [24, 25]. A recent review highlighted cfDNA’s advantages in population genetics, including large sample sizes, cost-effectiveness, and diverse research opportunities, while noting challenges related to its short fragment length and regional bias [26].

To date, no comprehensive studies have compared cfDNA and gDNA from the same group of participants, leaving differences in sequencing quality and variant detection unclear. To address this, we analyzed samples from 186 healthy individuals, sequencing both cfDNA and gDNA on the same platform. We compared data quality metrics, variant detection, allele frequency (AF) spectra, genotype concordance, population structure, and genomic association analysis performance.

Our results show that cfDNA has a higher duplication rate than gDNA in raw sequence files, leading to a lower effective sequencing depth after duplicate removal. However, at equivalent effective depths (∼37×), both DNA types exhibit highly comparable quality metrics. We also observed that bases with significant depth differences between cfDNA and gDNA were predominantly found in centromeric regions. While gDNA detected around 100K more single-nucleotide polymorphisms (SNPs) than cfDNA, both displayed nearly identical AF spectra, population structures, and high genotype concordance. Genomic association results were also highly consistent. The most notable difference was in insert size, influencing coverage, variant detection, and association signals for nonoverlapping SNPs. This study offers a comprehensive comparison of cfDNA and gDNA, highlight the potential role of cfDNA as an alternative to gDNA in genomic and genetic studies. We believe our findings will serve as a useful reference for researchers working in this field.

## Materials and methods

### Sample information

Participants in this study were recruited during their health examinations between 2021 and 2022 in Shenzhen. Informed consent was obtained from all participants prior to enrollment. The study received approval from the Institutional Review Boards of the Bioethics and Biosafety of BGI (BGI-IRB 21157). For each participant, 5 ml of blood was drawn and processed to separate white blood cells and plasma. The white blood cells were used to extract and sequence cellular gDNA, while the plasma was utilized to extract and sequence cfDNA.

### Quantitative traits

Upon recruitment, participants completed a questionnaire that included their date of birth, gender, ethnicity, province of origin, medication use, and other demographic information. Additionally, three anthropometric measurements—height, weight, and body mass index—were recorded. A 5 ml blood sample was collected from each participant and analyzed for various biochemical indicators at BGI-GBI Biotech. These indicators were categorized into five groups: liver-related (*n* = 7), kidney-related (*n* = 3), lipid-related (*n* = 4), protein-related (*n* = 4), and glucose levels (*n* = 1). Detailed information on these 22 traits is provided in Table 1.

**Table 1.** Summary of variant counts in cfDNA and gDNA

	cfDNA	gDNA	Overlap
Samples	186	186	186
Records	17 680 361	17 770 791	16 566 571
SNPs	14 963 735	15 068 657	14 278 527
Insertions and deletions (INDELs)	2838 856	2829 511	2395 767
Multiallelic sites	1151 672	1098 680	974 172
Multiallelic SNP sites	43 232	43 297	37 922
Ti/Tv	2.05	2.05	-

Single-cell sequencing libraries were prepared using the DNA Nanoball (DNB) elab C4 scRNA Preparation Kit (MGI). Sequencing data were processed with the Open Source Pipeline (https://github.com/MGI-tech-bioinformatics/DNBelab_C_Series_HT_scRNA-analysis-software) and analyzed using Scanpy (v1.10.4) [27]. We used Scrublet (v0.2.3) [28] to identify doublets in each library. Scrublet simulates doublets based on the observed data and calculates a doublet score for each single cell using a k-nearest neighbor classifier. Briefly, we first created an AnnData object using the raw count data from each library. Next, we ran the Scrublet function with the following parameters: expected_doublet_rate = 0.06, min_counts = 3, min_cells = 3, log_transform = True, min_gene_variability_pctl = 85, and n_prin_comps = 30. The “call_doublets” function was then applied with a threshold = 0.2, and the doublet detection results were added to the metadata for further analysis. Quality control was performed to exclude cells with gene counts outside 500–6000, total counts outside 1000–25 000, or mitochondrial gene percentages exceeding 10%. To account for sequencing depth variability, data were log-transformed using the ‘normalize_total’ function, and the top 2000 variable genes were identified with ‘highly_variable_genes’. Principal component analysis (PCA) of these genes was conducted, with the top 20 PCs used for Uniform Manifold Approximation and Projection to cluster cells in two dimensions. Batch effects were corrected with the ‘harmony_integrate’ function. Cellular identity was assigned by identifying cluster-specific differentially expressed genes and comparing them to known markers, resulting in the annotation of five cell subpopulations: B cells, CD4 + T cells, CD8 + T cells, myeloid cells, and innate lymphoid cells (ILCs).

### Sequencing and genotyping

Both cellular gDNA and plasma cfDNA were subjected to whole genome sequencing using the DNBSEQ platform with a paired-end 100 base pair (bp) mode and a targeted sequencing depth of ∼35×. A total of 186 participants provided both cellular gDNA and plasma cfDNA samples. The average original sequencing depths for gDNA and cfDNA were 62.55× and 47.34×, respectively. For each participant, the higher sequencing depth was down-sampled to match the lower depth to ensure a fair comparison. The raw sequencing data were stored in FASTQ (.fq) files. Quality control analysis was performed using the fastp software [29], which included the removal of adapter sequences and low-quality sequence fragments (reads). After quality control, we assessed and compared various sequence quality metrics, such as sequencing quality score, Q20, Q30, GC content, and insert size.

### Reads alignment

We employed Burrows-Wheeler Alignment tool (BWA) [30] to align the quality-controlled reads to the hg38 (GRCh38) reference genome [31], converting the aligned reads to BAM format and subsequently sorting them. Duplicate reads were removed from the sorted BAM files using SAMtools’ rmdup tool [32]. Base quality score recalibration (BQSR) was performed on the sorted BAM files using the GATK BaseRecalibrator [33] and known site information. Further BQSR and sorting were carried out using the GATK ApplyBQSR tool, which also generated index files. Comprehensive statistics for the calibrated BAM files, including contamination rate (FREEMIX), mapping rate, mismatch rate, unique rate, depth distribution of bases, and coverage rates at 1×, 10×, 20×, 30×, 40×, and 50×, were then generated using SAMtools [34] stats and VerifybamID [35].

### Individual-level variant detection

Individual-level variant calling involves identifying genetic variations in an individual’s genome relative to a reference sequence. We employed GATK HaplotypeCaller [36] to detect variants from the BAM files for each sample, generating gVCF (genomic variant call format) files that include sequencing information for both variant and nonvariant positions. We then used GATK GenotypeGVCFs to perform genotyping on single sample, this process yielded the genetic variations of each individual, stored in VCF files. After genotyping, we performed variant quality score recalibration (VQSR) using GATK VariantRecalibrator. Subsequently, for each individual, we calculated and compared several metrics: the number of SNPs, the number of INDELs, the heterozygosity to homozygosity (het/hom) ratio, and the transition to transversion (ti/tv) ratio.

### Population-level variant detection

Population-level variant calling aims to identify and analyze genetic variants across multiple individuals within a population [37]. We used GATK GenomicsDBImport to combine individual-level genotype files (gVCF) for joint genotyping with GenotypeGVCFs. Following this, we performed VQSR using GATK VariantRecalibrator to obtain population-level genetic variations stored in VCF files. To compare the genetic variations of cfDNA and gDNA at the population level, we assessed various metrics: the number of variant records, the number of SNPs, the number of INDELs, SNP density, population structure through PCA, the distribution of minor AF, concordance, and Pearson’s correlation. PCA was conducted using PLINK2 [38] with the “–pca” argument.

### Genomic association analysis

In this section, we evaluated the performance of the two DNA types in genomic association analyses using two categories of quantitative traits: regular phenotypes (Summarized in Supplementary Table S1) and single-cell RNA-seq (scRNA-seq) expression data. For regular phenotypes, we conducted GWAS, while for scRNA-seq expression data, we performed expression quantitative trait locus (eQTL) analysis.

GWAS are widely used to identify genetic variants, particularly SNPs, associated with complex traits and diseases [39]. Over the past two decades, >7000 GWASs have successfully identified significant SNPs linked to thousands of phenotypes [40]. Most GWAS studies are conducted using genotype data derived from gDNA sequencing, while only a few have utilized cfDNA genotype data. In this section, we compare the GWAS performance of gDNA and cfDNA. Specifically, we used 22 previously mentioned traits as phenotype data and conducted GWAS using genotype data from gDNA and cfDNA, respectively. Covariates included age, gender, and the top five principal components (PCs) of the corresponding genotype data [39, 41]. GWAS analysis was performed using PLINK 2.0 [38], with identical arguments and parameters applied to both gDNA and cfDNA. Key arguments included “–glm” to fit a generalized linear model, “–pheno-quantile-normalize” to normalize the phenotype data, and “–covar-variance-standardize” to standardize covariate data. Only SNPs with a minor allele frequency (MAF) > 0.05 [42], Hardy–Weinberg equilibrium (HWE) *P*-values >1e-5, and genotype missing rates <10% were included in the analysis.

eQTL analysis identifies genetic variants significantly associated with the expression of one or more genes [43]. Over the past decade, eQTL summaries have been widely used to interpret GWAS signals through transcriptome-wide association studies [44, 45]. To compare the eQTL analysis performance of cfDNA and gDNA, we used five cell subpopulations, including B cells, CD4 + T cells, CD8 + T cells, myeloid cells, and ILCs. Using TensorQTL [46, 47], we conducted *cis-*eQTL analysis by regressing scRNA-seq expression data on cfDNA and gDNA, respectively. Briefly, genes present in fewer than 90% of samples within each cell type were excluded. The remaining pseudobulk gene expression underwent inverse normal transformation across samples and were subsequently used as phenotype inputs in TensorQTL. The covariates included sex, age, the first two genotype PCs, and 50 PEER factors. PEER factors were derived from the top 2000 highly variable genes; for cell types with fewer than 2000 genes, all available genes were included. For cis-eQTL analysis, we focused on variants located within 1 Mb upstream or downstream of the gene’s transcription start site. We employed the argument “–mode *cis*” with a MAF threshold set to 0.01. The “map_nominal” function was used to derive nominal *P*-values for each variant-gene. Subsequently, the “map_*cis*” function was applied to conduct 10 000 permutations, generating phenotype-level summary statistics and empirical *P*-values. This approach enabled the calculation of genome-wide false discovery rate (FDR) (q-value) for robust statistical inference.

## Results

**Figure 1.**
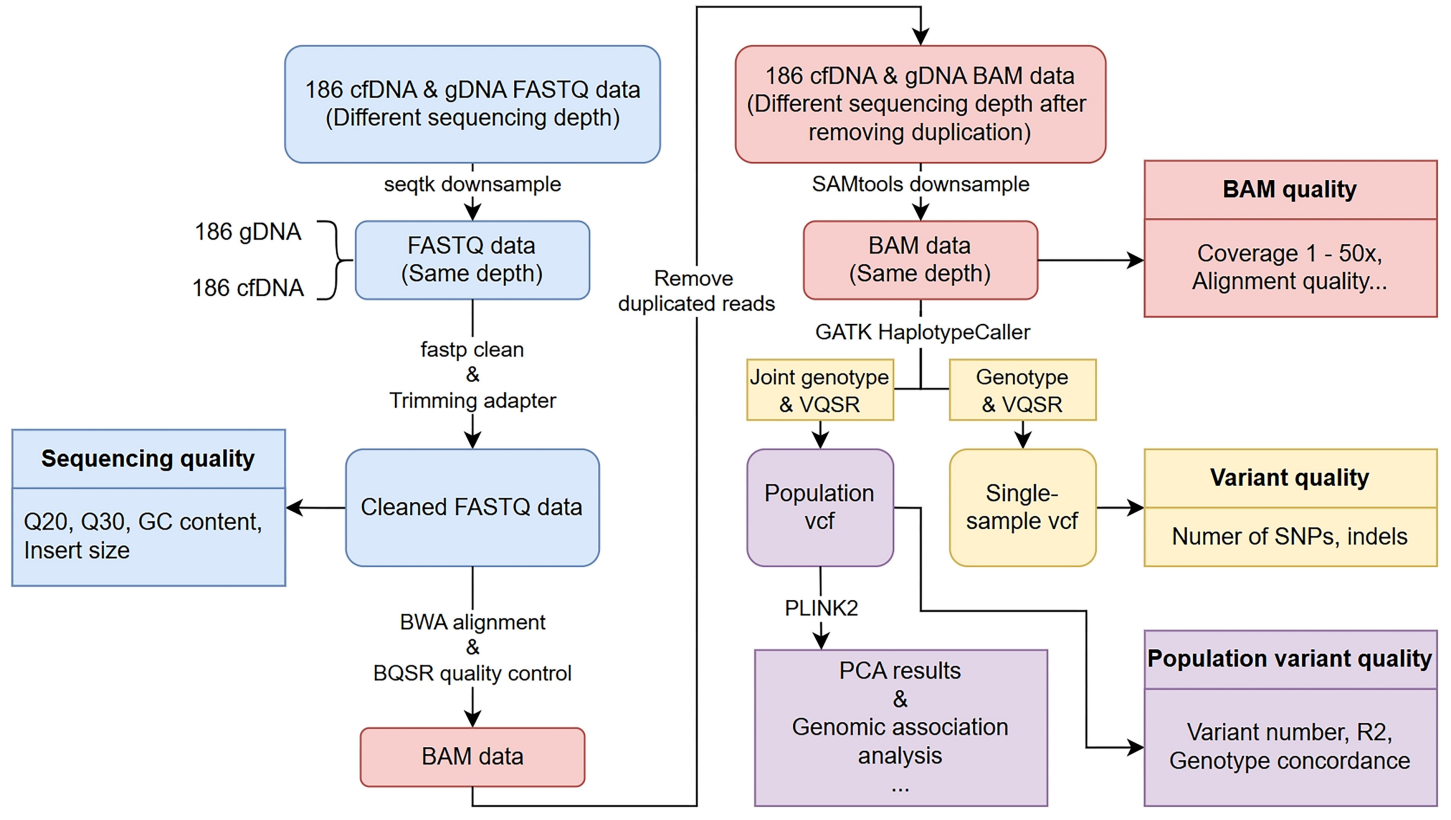
Study workflow. Blue, red, yellow, and purple were used to represent the processes of FASTQ quality control, BWA alignment, individual- and population-level variant detection, and genomic association analysis, respectively.

We comprehensively compared the performance characteristics of two distinct DNA sequencing methodologies across various stages of sequencing and analysis. Figure 1 illustrates the complete workflow and comparative metrics employed in this research.

### Sample information

In our study, we sequenced both cfDNA and cellular gDNA from 186 participants. Detailed demographic data, including age, gender, ethnicity, and place of origin were collected and are presented in Supplementary Fig. S1A–D. In summary, the male-to-female ratio among participants is ∼1:1. Participants aged between 20 and 29 accounted for 59% of the total, with the majority being of Han ethnicity. The participants’ places of origin spanned 25 provinces and cities.

### Sequence depth

The average raw sequencing depths for cfDNA and gDNA are 47.34× and 62.55×, respectively (Supplementary Fig. S2A). To enable fair comparisons, we down-sampled the raw sequence data (in .fq format) with higher depth to match the lower depth for each participant. Following this adjustment, the two types of DNA achieved identical sequencing depths of 46.73× (Supplementary Fig. S2B).

### Sequence features

**Figure 2.**
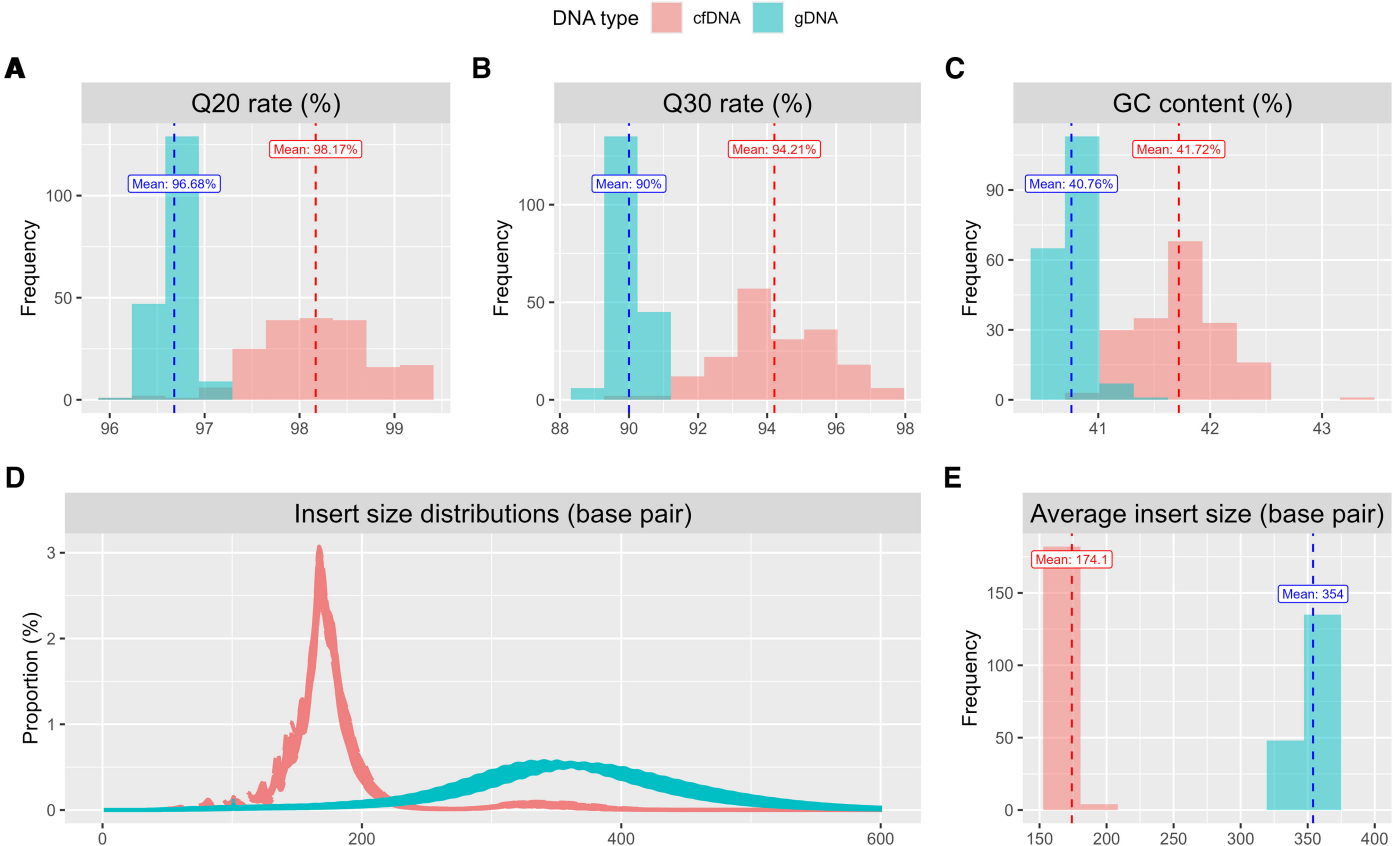
Comparisons of sequencing quality metrics between cfDNA and gDNA. (**A**) Q20 rate for cfDNA and gDNA, (**B**) Q30 rate for cfDNA and gDNA, (**C**) GC content for cfDNA and gDNA, (**D**) insert size distributions of cfDNA and gDNA, and (**E**) average insert size for cfDNA and gDNA.

From the cleaned fastq files after quality control, we obtained several sequence features, including fastq quality score, Q20, Q30, GC content, and insert size. The quality score was calculated using the formula −10 × log_10_(p), where p represents the probability of error. Overall, the FASTQ quality scores for both cfDNA and gDNA exceeded 30, indicating high-quality sequencing data (Supplementary Fig. S2C). Notably, the quality scores of cfDNA were consistently higher than those of gDNA across DNA fragments. This difference is likely attributable to batch effects, as cfDNA and gDNA were sequenced in separate batches. However, this should not be interpreted as a general trend. Q20 (a quality score of 20) and Q30 (a quality score of 30) represent the percentages of bases with quality scores >20 and 30, respectively, and follow the overall quality score pattern. Notably, both cfDNA and gDNA achieved > 95% Q20 and > 85% Q30 (Fig. 2A and B), demonstrating high-quality sequencing data for both DNA types on the BGISEQ platform [48, 49]. Regarding GC content, the averages were 41.72% [standard deviation (SD) = 0.3%] for cfDNA and 40.76% (SD = 0.1%) for gDNA (Fig. 2C). The ideal GC content in human genomes is around 41% [49, 50] within a range of 39%–43%, indicating both cfDNA and gDNA fall within normal values.

For insert sizes across all samples, we present both the full distributions and the distribution of sample averages (Fig. 2D and E). Both distributions indicate that the average insert sizes for cfDNA and gDNA are ∼170 and 350 bp, respectively. gDNA originates from the complete genome of white blood cell nuclei, with DNA fragments generated through physical fragmentation during the sequencing process. On the DNBSEQ platform, the typical insert size for gDNA in short-read paired-end whole genome sequencing libraries is ∼350 bp [51]. In contrast, cfDNA consists of naturally short DNA fragments (∼167 bp), primarily released from apoptotic cells [52, 53], and is not subjected to physical shearing during sequencing.

In summary, both cfDNA and gDNA exhibit high sequencing quality across various metrics, with cfDNA showing the expected shorter insert size compared to gDNA.

### Reads alignment and second down-sampling

After BWA alignment and BQSR quality control, we generated aligned sequence data in BAM format. The average duplication rates were calculated as 18.63% for cfDNA and 1.14% for gDNA, with ranges of 7.62%–36.28% and 0.60%–1.94%, respectively (Supplementary Fig. S3A). The high duplication rate of cfDNA is attributed to the relatively small quantity of DNA extracted. To meet the DNA requirements for sequencing, more cycles of polymerase chain reaction (PCR) are required, which introduce multiple copies of identical DNA fragments. Consequently, duplicated reads must be removed during downstream analysis, leading to a reduced effective sequencing depth for cfDNA. After duplicated reads were removed, the sequencing depths for cfDNA and gDNA were 37.72× and 45.98×, respectively (Supplementary Fig. S3B). To achieve comparable sequencing depths between cfDNA and gDNA for each participant after removing duplicated reads, we performed a second round of down-sampling, adjusting the deeper gDNA data to match the lower sequencing depth of cfDNA. Following this second down-sampling, cfDNA and gDNA exhibited nearly identical sequencing depths for each participant (Supplementary Fig. S3C).

### Post-alignment metrics

From the depth-matched BAM files of cfDNA and gDNA, we compared several metrics: the sequence-only estimate of contamination (measured by FREEMIX), mapping rate, mapping quality, mismatch rate, depth distribution of bases, and coverage at different sequence depths. The average FREEMIX values for cfDNA and gDNA were 0.16% and 0.34%, respectively (Supplementary Fig. S4A), both well below the generally acceptable contamination threshold of 5% [54, 55]. The mapping rates averaged 99.87% for cfDNA and 99.92% for gDNA (Supplementary Fig. S4B), consistent with the DNBSEQ PE-100 platform, which typically exceeds 99% [56]. The mapping quality score quantifies the likelihood of a read being incorrectly placed and is calculated based on sequence quality [57]. Accordingly, the mapping quality is expected to follow a similar pattern to sequence quality. Specifically, the mapping quality scores for cfDNA and gDNA were 34.04 and 32.76, respectively (Supplementary Fig. S4C). The mismatch rate, defined as the number of reads with specific mismatch patterns (e.g. A→C, A→G, and G→T) relative to the total number of aligned reads [58], averaged 0.40% for cfDNA and 0.65% for gDNA (Supplementary Fig. S4D)—both well within the acceptable mismatch rate of <1% [49].

We further analyzed the depth distribution of bases across the 22 chromosomes for cfDNA and gDNA. We divided the genome into windows of 10 000 bp and calculated the average depth of all bases within each window. The resulting distributions revealed greater variability in cfDNA base depths, whereas gDNA base depths were more consistently distributed (Supplementary Fig. S5A). Next, we calculated the depth difference between cfDNA and gDNA for each window and defined a window as significantly different if the depth difference exceeded three standard deviations from the mean (Supplementary Fig. S5B). We identified 756 such windows with significant differences. Upon annotating the starting sites of these 756 windows using the track data downloaded from UCSC Genome Browser (https://genome.ucsc.edu/), we found that the majority (707, 93.5%) were located in the sub-table “Difficult regions” of track “GIAB Problematic Regions,” with most of these (513/707) positioned in centromeres. The remaining windows included in track “Gap” (16) and uncategorized regions (33) (Supplementary Fig. S6).

**Figure 3.**
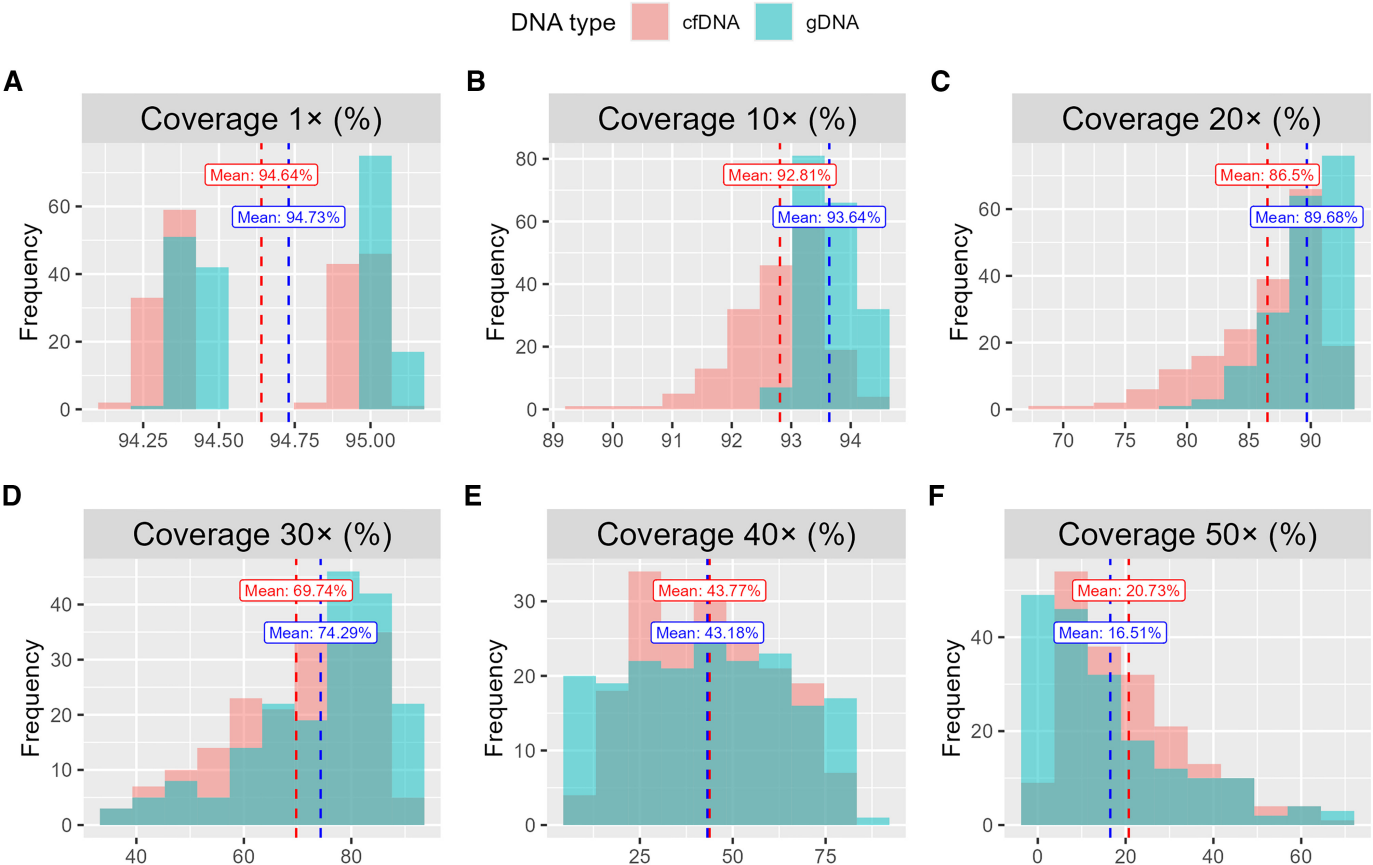
Comparison of coverage across different sequencing depths. (**A**)–(**F**) Coverage at sequencing depths of 1×, 10×, 20×, 30×, 40×, and 50×, respectively.

Coverage at a specific depth (e.g. 1×, 10×) refers to the percentage of bases in the genome that have been sequenced to at least that depth. For example, a coverage of 90% at 1× means that 90% of the bases in the genome have been sequenced to a depth of at least 1×, while the remaining 10% have lower coverage or no coverage. Higher coverage indicates that a larger proportion of bases have been sequenced to meet or exceed the specified depth threshold. For both cfDNA and gDNA, coverages at 1×, 10×, 20×, 30×, 40×, and 50× were computed and compared (Fig. 3). Notably, for sequencing data with a depth of ∼30×, coverage at 20× is typically the primary focus, while higher depths receive less emphasis in many analyses. Our goal is to identify coverage patterns across different sequencing depths between cfDNA and gDNA, thus we compared coverages at various depths both below and above 30× .

We observed that at depths below 30×, gDNA consistently exhibits slightly higher coverage than cfDNA, with an average difference of 1.37%. Specifically, at 1×, 10×, and 20×, both cfDNA and gDNA achieve coverage rates of ∼95%, over 90%, and over 85%, respectively. This indicates that for both DNA types, a high percentage of target bases are successfully sequenced. However, at 30× depth, coverage drops below 75% for both cfDNA and gDNA, with gDNA maintaining a slight advantage (74.29% versus 69.74%). At 40× depth, the coverage rates for cfDNA and gDNA are nearly identical, at 43.77% and 43.18%, respectively, with cfDNA showing a marginally higher coverage (0.59%). By 50× depth, cfDNA coverage surpasses that of gDNA more notably, at 20.73% compared to 16.51% (a difference of 4.22%).

In summary, at sequencing depths below 40× (or 37×, the average depth for cfDNA and gDNA in this dataset), gDNA consistently demonstrates higher coverage than cfDNA. Conversely, at depths above 40×, cfDNA coverage exceeds that of gDNA. These findings suggest that across the genome, the distribution of base depths is more uniform in gDNA compared to cfDNA.

To compare the coverage deviation between the two DNA types, we derived the probability density function (PDF) for their coverage distributions. In this analysis, $n$ represents the size of the genome to be sequenced, $L$ denotes the insert size of the DNA fragment, $r$ indicates the sequencing depth (here, 100 bp), and $k$ specifies the read counts. For cfDNA and gDNA, we assumed that their insert sizes were <200 bp and >200 bp, respectively (Supplementary Fig. S7). Consequently, we derived their PDFs separately. For simplicity, we calculated the probability mass function (PMF) for the coverage distribution when the read count equals 1 ($k = 1$). Let ${{x}_1}$ and ${{x}_2}$ denote the random variable for cfDNA coverage and gDNA coverage, respectively; the resulting PMFs for cfDNA and gDNA were as follows:


\begin{eqnarray*}
{\rm PMF\ of\ cfDNA\ coverage} &=& f\left( {{{x}_1},\ {{p}_1}} \right)\nonumber\\ &=& \left\{ {\begin{array}{@{}*{1}{c}@{}} {1 - \frac{L}{n}\ if\ {{x}_1} = 0}\\ {\frac{L}{n}\ if\ {{x}_1} = \frac{{2r}}{L}} \end{array}} \right.,\ {\rm where}\ {{p}_1} = \frac{L}{n}
\end{eqnarray*}

\begin{eqnarray*}
{\rm PMF\ of\ gDNA\ coverage} &=& f\left( {{{x}_2},{{p}_2}} \right)\nonumber\\ &=& \left\{ {\begin{array}{@{}*{1}{c}@{}} {1 - \frac{{2r}}{n}\ if\ {{x}_2} = 0}\\ {\frac{{2r}}{n}\ if\ {{x}_2} = 1} \end{array}} \right.,\ {\rm where}\ {{p}_2} = \frac{{2r}}{n}
\end{eqnarray*}

Based on the formulas above, we derived the expected value and variance for cfDNA and gDNA coverage as follows:


\begin{eqnarray*}
E\left( {{{x}_1}} \right) = \frac{{2r}}{n},\ Var\left( {{{x}_1}} \right) = 4{{r}^2}\left( {\frac{1}{{Ln}} - \frac{1}{{{{n}^2}}}} \right)
\end{eqnarray*}

\begin{eqnarray*}
E\left( {{{x}_2}} \right) = \frac{{2r}}{n},\ Var\left( {{{x}_2}} \right) = \frac{{2r}}{n}\left( {1 - \frac{{2r}}{n}} \right)
\end{eqnarray*}

We calculated the difference in variance between cfDNA and gDNA coverage as follows:


\begin{eqnarray*}
Var\left( {{{x}_1}} \right) - Var\left( {{{x}_2}} \right) = \frac{{2r}}{n}\left( {\frac{{2r}}{L} - 1} \right) > 0
\end{eqnarray*}

Thus,


\begin{eqnarray*}
Var\left( {{{x}_1}} \right) > Var\left( {{{x}_2}} \right)
\end{eqnarray*}

This explains why the cfDNA coverage distribution is broader compared to that of gDNA.

### Individual-level variant detection

Using individual-level variant data stored in VCF files, we compared several metrics, including variant depth, Ti/Tv ratio, Het/Hom ratio, the number of SNPs, and the number of INDELs. The variant depth for both cfDNA and gDNA was ∼35× (Supplementary Fig. S8A), slightly lower than the average sequencing depth of 37× at the individual level. The average Ti/Tv ratio was 2.02 for cfDNA and 2.01 for gDNA (Supplementary Fig. S8B), indicating high-quality SNP calling, as the expected ratio for human whole-genome data typically ranges from 2.0 to 2.2 [59]. These values confirm the high quality of the detected variants in both cfDNA and gDNA. The average Het/Hom ratio was 1.33 for both cfDNA and gDNA (Supplementary Fig. S8C), which is within the normal range for Asians, where the median value is ∼1.4 [59]. This suggests normal heterozygosity levels in both cfDNA and gDNA.

**Figure 4.**
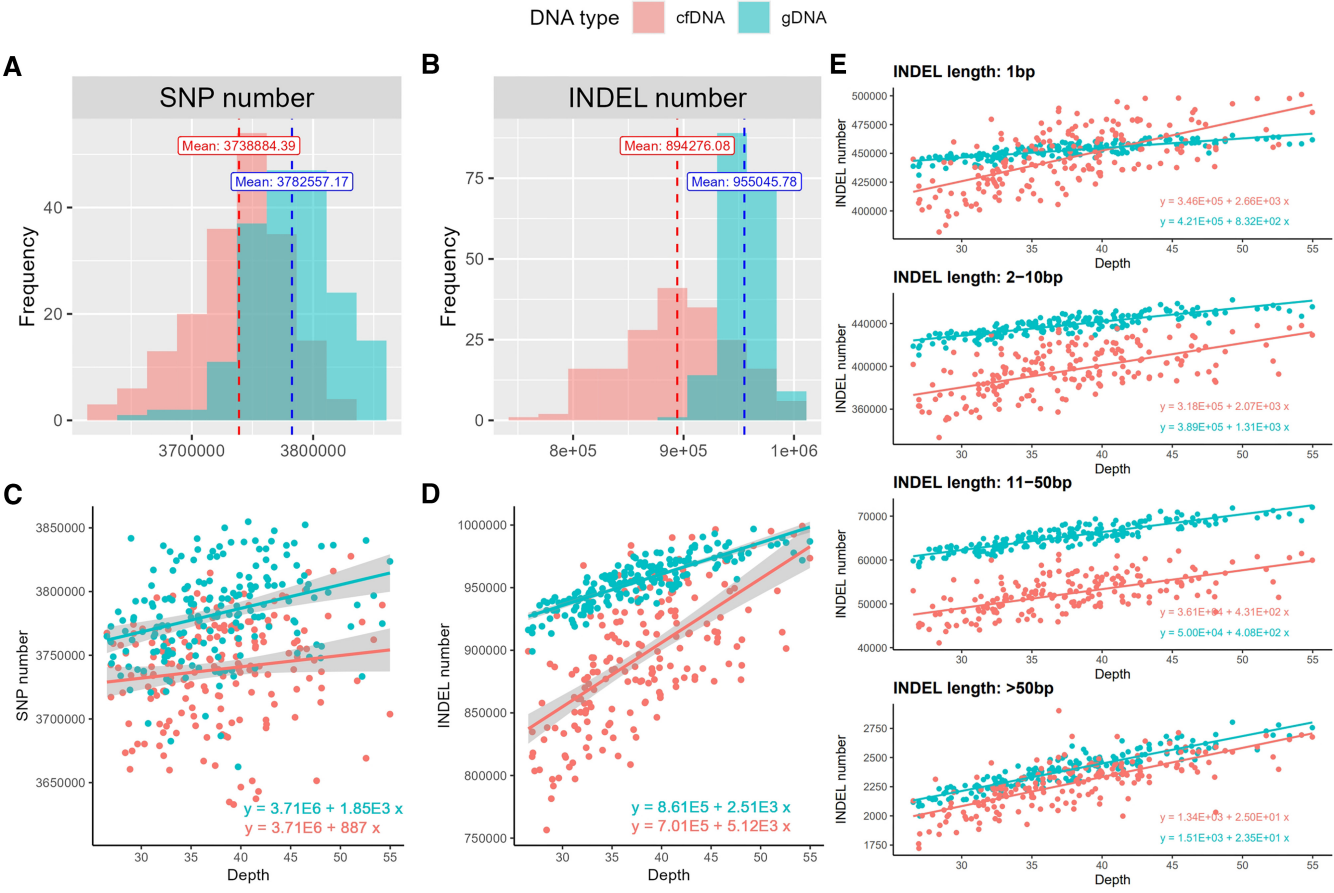
Comparison of variant counts. (**A**) Number of identified SNPs in cfDNA and gDNA; (**B**) number of identified INDELs in cfDNA and gDNA; (**C**) number of SNPs in cfDNA and gDNA across increasing sequencing depths; and (**D**) number of INDELs in cfDNA and gDNA across increasing sequencing depths; (**E**) Number of INDELs in cfDNA and gDNA across increasing sequencing depths, stratified by INDEL length

On average, cfDNA and gDNA contained 3.74 million and 3.78 million SNPs, respectively (Fig. 4A). The average number of INDELs was 0.89 million for cfDNA and 0.96 million for gDNA (Fig. 4B). For both types of variants, gDNA detected more than cfDNA. To the best of our knowledge, no previous studies have directly compared or reported the number of identified variants, including SNPs and INDELs, for cfDNA and gDNA at the same sequencing depth at both individual and variant levels. In this study, we observed that gDNA identified more SNPs and INDELs than cfDNA. We hypothesize that this may represent a general trend, as gDNA libraries have longer insert sizes compared to cfDNA, and longer insert sizes are associated with improved variant detection performance [60, 61].

For each sample, we plotted the number of identified SNPs and INDELs as sequencing depth increased. The number of SNPs showed a slight upward trend, with linear slopes of 887 and 1850 for cfDNA and gDNA, respectively (Fig. 4C). In contrast, INDELs exhibited a steeper increase, with linear slopes of 5120 and 2510 for cfDNA and gDNA, respectively (Fig. 4D). To further understand this phenomenon, we categorized INDELs into four length groups: 1 bp, 2–10 bp, 11–50 bp, and >50 bp (Fig. 4E). We found that only the 1bp INDELs showed a significant difference in slopes between the two groups. Notably, a sequencing depth of 30× is sufficient to capture most SNPs, with limited gains from deeper sequencing. However, shorter INDELs continue to increase due to their detection challenges and can benefit more from higher sequencing depth, especially for cfDNA.

### Population-level variant detection

In this section, we performed population-level variant detection. Consistent with the individual-level results, gDNA identified more variants, including both SNPs and INDELs, than cfDNA, with a high percentage of variants shared between the two. Specifically, gDNA identified over 17.77 million variants, comprising 15.07 million SNPs and 2.83 million INDELs, while cfDNA identified ∼17.68 million variants, including 14.96 million SNPs and 2.84 million INDELs (Table 1). The population-level Ti/Tv ratio was identical for cfDNA and gDNA at 2.05, reflecting the high quality of variant detection in both datasets.

**Figure 5.**
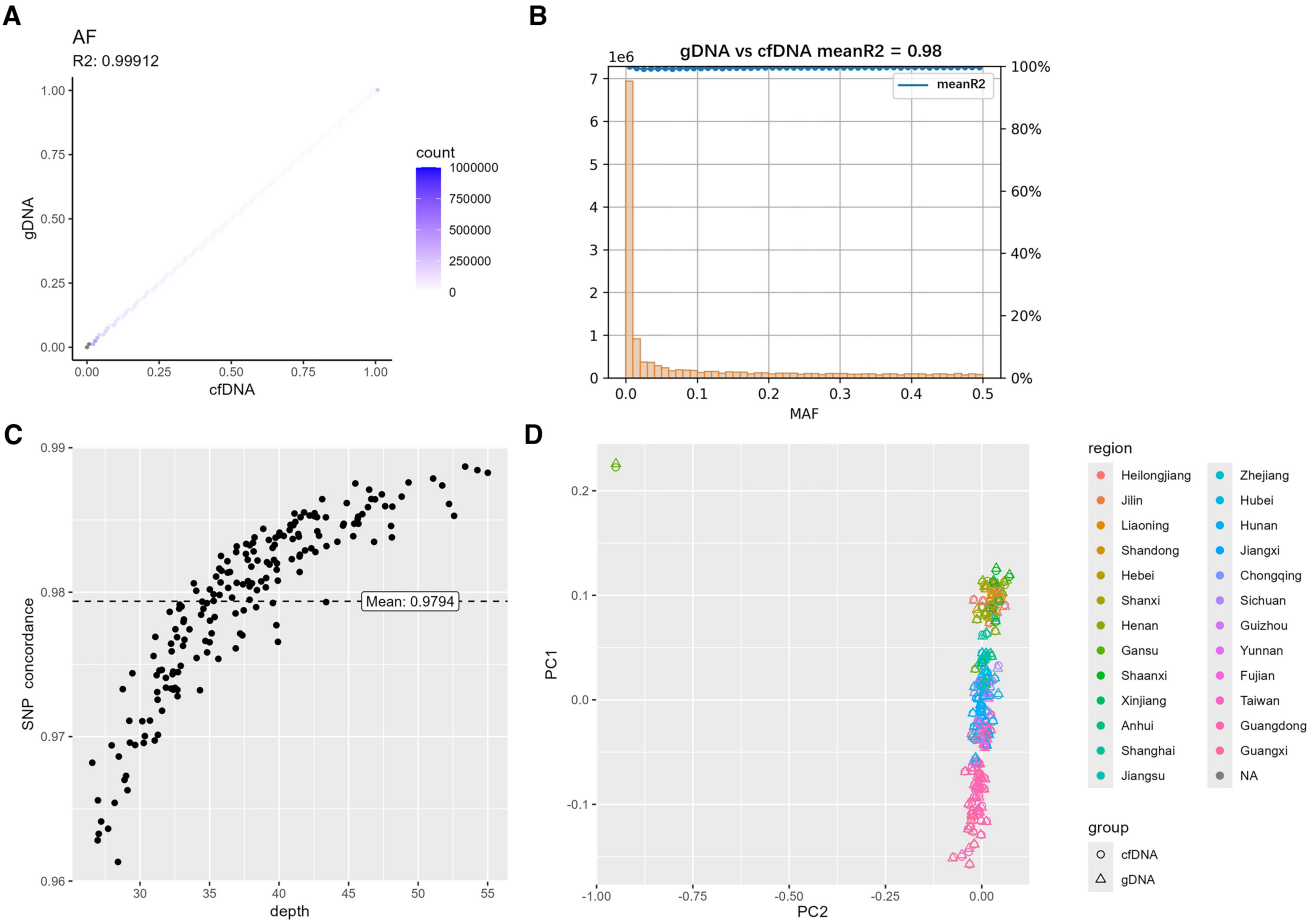
Comparison of two DNA types: AF spectrum, genotype values, and population structure. (**A**) Scatter plot of AF for SNPs in cfDNA and gDNA; (**B**) distribution of overlapping SNPs across different MAF intervals between cfDNA and gDNA, along with the average squared Pearson correlation coefficients of genotype values in each interval; (**C**) genotype concordance between cfDNA and gDNA across 186 participants; and (**D**) PC1–PC2 scatter plot comparing cfDNA and gDNA.

For the 16.6 million overlapping SNPs shared between cfDNA and gDNA, we calculated the MAFs in each dataset separately and plotted a scatter plot comparing the MAFs of the two DNA types. The squared Pearson correlation coefficient (R²) for MAFs between cfDNA and gDNA was 0.999 (Fig. 5A), indicating that the AF spectrums derived from the two DNA types are nearly identical. We also examined the correlation of genotype values between cfDNA and gDNA across different MAF intervals. All shared SNPs were grouped into MAF intervals with increments of 0.01. For each SNP within an interval, we calculated the R² of genotype values between cfDNA and gDNA across all individuals and then averaged the R² values for SNPs within that interval. These averages were plotted in Fig. 5B, with the overall mean correlation across intervals being 0.98. This result demonstrates a high consistency of genotype values between cfDNA and gDNA for both rare and common variants.

In addition, we assessed the site-level concordance between the two DNA types using GATK. Specifically, for each individual, concordance was calculated as the ratio of SNPs with identical genotype values between the two DNA types to the total number of overlapping SNPs. The scatter plot showing concordance across all 186 individuals is presented in Fig. 5C. The average concordance was 0.979, with values ranging from 0.961 to 0.989, indicating a high degree of consistency in genotype values between the two DNA types in the population.

We performed PCA on the genotype data from cfDNA and gDNA and generated a PC1–PC2 scatter plot for all individuals (Fig. 5D). Different dot shapes (circles for cfDNA and triangles for gDNA) were used to represent DNA types, while colors indicated the individuals’ places of origin. Notably, for each individual, the cfDNA and gDNA data points were almost perfectly overlapped, demonstrating the high consistency of population structure inferred from cfDNA and gDNA. PC1 primarily reflects the latitudinal geographical location, as indicated by the color-coded provinces of origin. One outlier was identified whose place of origin is Gansu, located in the Hexi Corridor, a historically significant commercial hub on the Silk Road connecting China to the West since the Han Dynasty [62]. This individual was excluded from subsequent GWAS.

In summary, based on population-level variant detection, we concluded that cfDNA and gDNA identified comparable numbers of SNPs, with gDNA detecting slightly more. The consistency of PCA and AF indicated that the batch effects were minimal between the two datasets, as evaluated by the methods recommended by previous studies [63, 64]. Through analyses of AF spectra, genotype values, and population structure, we demonstrated the high genotype consistency between cfDNA and gDNA.

### Genomic association analysis

In this section, we conducted association analyses between the two DNA types and two categories of quantitative traits: regular phenotypes and scRNA-seq expression data. For regular phenotypes, we performed GWAS using PLINK 2.0 [38]. For scRNA-seq expression data, we carried out eQTL analysis using TensorQTL [46, 47].

**Figure 6.**
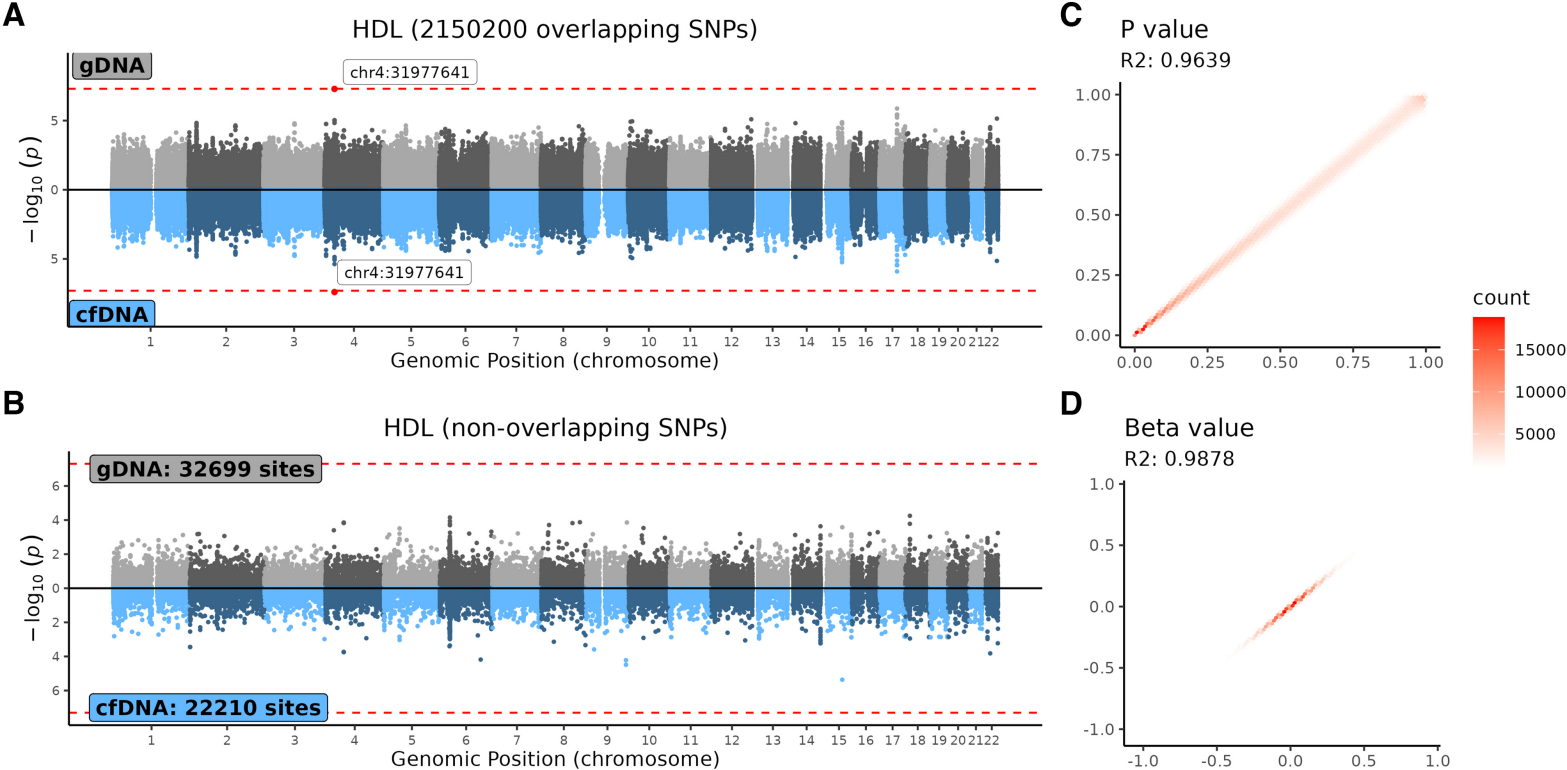
GWAS performance comparison of cfDNA and gDNA in HDL trait. (**A**) Mirrored Manhattan plot for GWAS of high-density lipoprotein (HDL) cholesterol levels based on 2150 200 overlapping SNPs between cfDNA and gDNA; (**B**) Manhattan plots for GWAS of HDL levels using 32 699 unique SNPs from the gDNA dataset (top) and 22 210 unique SNPs from the cfDNA dataset (bottom); (**C**) scatter plot of −log_10_(*P*-values) for overlapping SNPs in GWAS results between cfDNA and gDNA; and (**D**) scatter plot of beta values for overlapping SNPs in GWAS results between cfDNA and gDNA.

Given the relatively small sample size of 185 participants, the GWAS analysis identified only a few genome-wide significant SNPs across all 22 phenotypes using genotype data from both cfDNA and gDNA. After masking SNPs in repeated region [65], filtering SNPs with MAF < 0.05, HWE *P*-value < 1e-5, and genotype missing rate >10%, 172 410 and 2182 899 SNPs remained for cfDNA and gDNA, respectively, with 2150 200 SNPs overlapping between the two. Overall, the GWAS results based on cfDNA and gDNA were highly consistent, as evidenced by the Manhattan plots, scatter plots of *P*-values, and scatter plots of beta values (Fig. 6 and Supplementary Fig. S9). The squared Pearson correlation coefficients (R²) for *P*-values and beta values of overlapping SNPs between the two DNA types averaged 0.967 and 0.989, respectively, across all 22 phenotypes. For nonoverlapping SNPs, correlation coefficients could not be computed; however, mirrored Manhattan plots demonstrated high consistency at the same loci between cfDNA and gDNA GWAS results. We highlighted the comparison results for exemplar phenotypes, such as high-density lipoprotein cholesterol (HDL-C) levels, in Fig. 6, with results for the remaining phenotypes presented in Supplementary Fig. S9.

**Figure 7.**
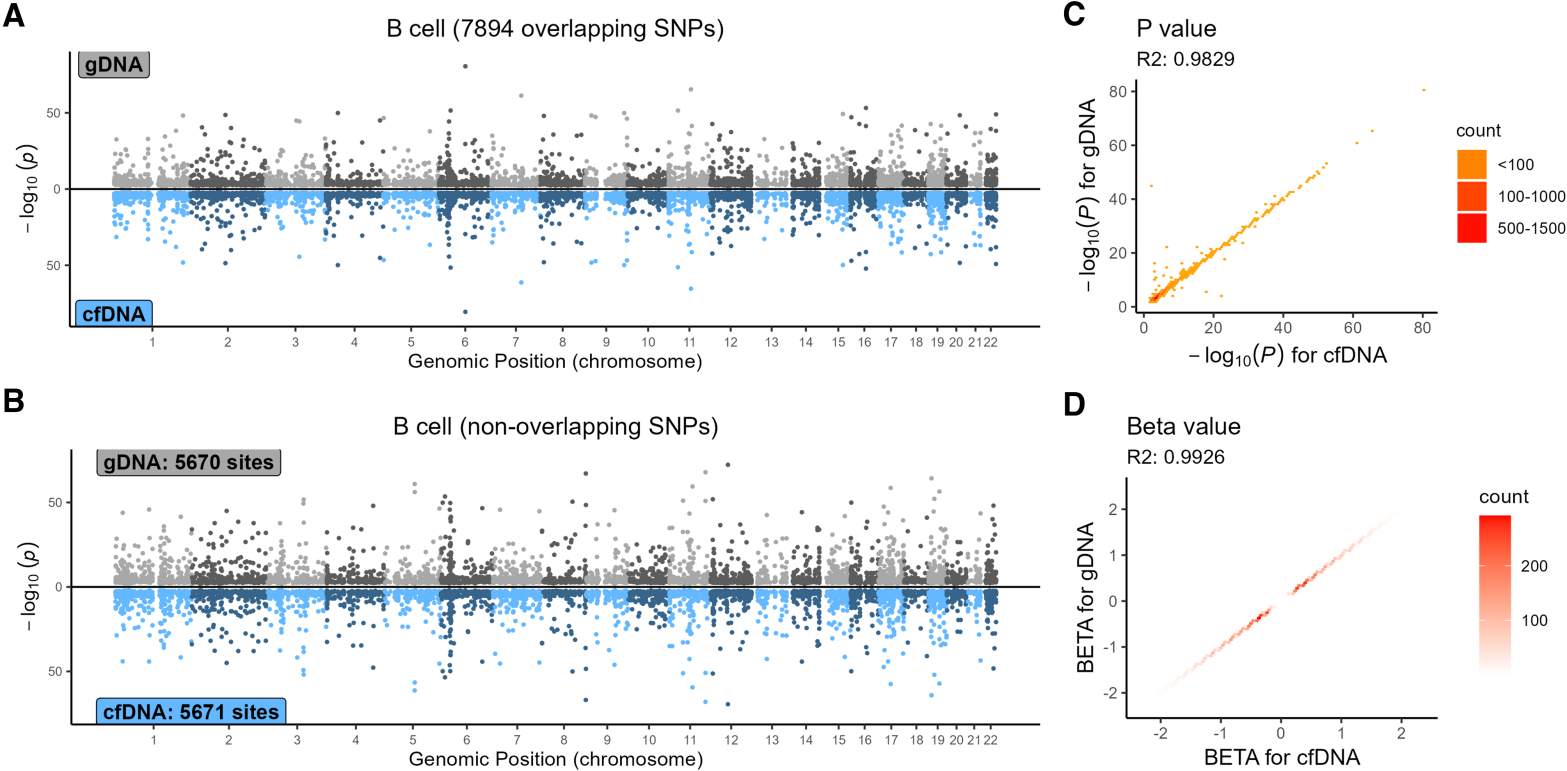
eQTL performance comparison of cfDNA and gDNA in B cells. (**A**) Mirrored Manhattan plot for eQTL analysis of B cells based on 7894 overlapping SNPs between cfDNA and gDNA; (**B**) Manhattan plots for eQTL analysis of B cells using 5670 unique SNPs from the gDNA dataset (top) and 5671 unique SNPs from the cfDNA dataset (bottom); (**C**) scatter plot of −log_10_(*P*-values) for overlapping SNPs in eQTL results between cfDNA and gDNA; and (**D**) scatter plot of beta values for overlapping SNPs in eQTL results between cfDNA and gDNA.

Similar to the GWAS comparison results, the eQTL analysis demonstrated high consistency between cfDNA and gDNA (Fig. 7 and Supplementary Fig. S10). Among the 186 participants with both cfDNA and gDNA, 179 also had scRNA-seq expression data. After masking SNPs in repetitive regions, 14 937 426 SNPs remained for cfDNA and 15 050 832 for gDNA, with 14 249 022 overlapping between the two. SNPs with a MAF < 0.01, located beyond ±1 MB from the *cis-*window, with a FDR > 0.05, or an effect size (beta) of 0 were excluded from the eQTL results. Consequently, the actual number of SNPs in the eQTL results was significantly lower than the total number of SNPs analyzed. Across five cell subpopulations (B cells, CD4 + T cells, CD8 + T cells, myeloid cells, and ILCs), the squared Pearson correlation coefficients for −log_10_(*P*-values) of overlapping SNPs ranged from 0.8756 to 0.9829, with an average of 0.9533. Similarly, the squared Pearson correlation coefficients for beta values of overlapping SNPs ranged from 0.9858 to 0.9926, averaging 0.9897. For nonoverlapping SNPs, mirrored Manhattan plots revealed high concordance in *P*-values at the same loci between cfDNA and gDNA.

In summary, the results of genomic association analyses, including GWAS and eQTL studies, consistently showed strong agreement between cfDNA and gDNA.

## Discussion

cfDNA and gDNA are two types of DNA that both carry the genetic information of the subject but differ in several key characteristics due to their origins. gDNA, derived from the nuclei of white blood cells, consists of long, intact DNA molecules, while cfDNA is fragmented DNA primarily released from apoptotic cells into body fluids. Two major differences between cfDNA and gDNA sequencing data are the rate of duplicated reads and insert sizes.

First, cfDNA data has a higher rate of duplicated reads compared to gDNA. This is a consequence of library construction, as the low cfDNA amount (∼10 ng/ml plasma) requires more PCR amplification cycles to produce sufficient material for sequencing. The necessary step of removing duplicated reads results in a lower effective sequencing depth for cfDNA. Therefore, to achieve an equivalent amount of usable sequencing data, cfDNA typically requires a higher raw sequencing depth than gDNA.

Second, the difference in insert sizes arises from their origins. gDNA begins as long, intact DNA strands that are physically or enzymatically sheared during library preparation to achieve a specific fragment length. In contrast, cfDNA originates as short DNA fragments naturally released from cells, eliminating the need for shearing. This inherent shortness in cfDNA insert sizes are strongly related to the uneven genomic coverage [66] and a smaller number of detected variants. In this study, we discussed the relevant aspects and found significant depth differences between the two DNA types, mainly in centromeric regions. This phenomenon may be due to the fact that centromeric regions themselves are highly repetitive sequences [67], and the analysis of these regions has always been a challenge in the field of short-read sequencing [68, 69]. We hypothesize that the performance of the two different insert sizes of DNA during alignment differs in similar repetitive regions, with gDNA having a higher proportion of correct alignments due to its larger fragment size, thus resulting in significant differences in the performance of the two materials in these regions. From this perspective, we speculate that the differences observed in variant detection (e.g. the number of INDELs varying with sequencing depth and the nonoverlapping SNPs in population genetic analysis) may also stem from alignment differences caused by different insert sizes. From previous works, we have also found similar conclusions: insert size affects the accuracy of variant detection, and longer insert sizes are more precise [70].

A unique characteristic of cfDNA is its ability to offer molecular insights beyond genetic information, including concentration [24], fragment size and patterns [71, 72], and epigenetic status [73], among others. These features serve as valuable biomarkers for monitoring and predicting physiological conditions. Depending on the research design and objectives, researchers may opt to sequence either cfDNA or gDNA.

Our study may have several aspects to be improved. First, the sample size of 186 is relatively small, even though it is large enough to obtain reliable results of the comparison of data quality metrics and variant detection, there are no well-established significant genome-wide signals associated with the studied 22 phenotypes. Therefore, we lack the conclusion for the GWAS performance comparison between cfDNA and gDNA on significant hits even though we expect nearly identical results based on the current nonsignificant associations. Second, for the current genomic association analysis, we investigate some general phenotypes (e.g. height, HDL-C) and scRNA-seq expression data, but no other omics data, for example, proteome data or epigenome data. Many studies have been conducted to investigate the associations between the genotype data with these omics data and obtained particular quantitative trait locus (QTL), such as pQTL (protein QTL) [74] and meQTL (methylation QTL) [75]. These association analyses help identify genetic variants that are associated with protein levels and DNA methylation, elucidating how genetic variations influence molecular and phenotypic traits from multiple perspectives. For a more comprehensive comparison between cfDNA and gDNA, we should have also performed these association analyses to see if there is a difference in identifying these QTLs.

## Lead contact

Further information and requests should be directed to and will be fulfilled by the lead contact, Huanhuan Zhu (zhuhuanhuan1@genomics.cn).

## Supplementary Material

lqaf119_Supplemental_File

## Data Availability

All data supporting the findings of this study are included within the manuscript. No additional individuallevel data are available for public access.
