# Supplementary material for: Cell-free DNA as a potential alternative to genomic DNA in genetic studies
Source: NAR Genom Bioinform. 2025 Sep 9;7(3):lqaf119. doi: 10.1093/nargab/lqaf119 (PMC12408905; doi:10.1093/nargab/lqaf119)
Supplement: lqaf119_Supplemental_File [file lqaf119_supplemental_file.pdf]

Table S1. Detailed information on GWAS traits.

Item_Name	Item_Name_Full	Item_catagory	Unit	RefenceValue	Sample size	Mean	sd
**Height**	Height	Anthropometry		-	186	165.63	8.46
**Weight**	Weight	Anthropometry		-	186	61.05	12.15
**BMI**	Body Mass Index	Anthropometry		-	186	22.12	3.18
**GLU**	Glucose	Glucose	mmol/L	3.9-6.1 mmol/L	183	5.03	0.55
**UR**	Urea	Kidney-related	mmol/L	3.4-12.3 μ mol/L	183	4.90	1.12
**CR**	Creatinine	Kidney-related	μ mol/L	Female: 44.2-88.4 μ mol/L; Male: 44.2-106.1 μ mol/L	183	67.08	12.96
**UA**	Uric acid	Kidney-related	μ mol/L	Female: 143-339 μ mol/L; Male: 202-416 μ mol/L	183	355.02	108.36
**CHOL**	Total cholesterol	Lipids	mmol/L	2.9-5.86 mmol/L	183	4.80	0.82
**HDL**	High-density lipoprotein cholesterol	Lipids	mmol/L	0.9-2.19 mmol/L	183	1.54	0.30
**LDL**	Low-density lipoprotein cholesterol	Lipids	mmol/L	Female: 0.44-1.17 mmol/L; Male: 0.51-1.72 mmol/L	183	2.73	0.59
**TG**	Triglycerides	Lipids	mmol/L	0-3.37 mmol/L	183	1.18	0.80
**ALT**	Alanine transaminase	Liver-related	U/L	0-40 U/L	183	27.77	14.08
**AST**	Aspartate transaminase	Liver-related	U/L	0-40 U/L	183	34.15	8.35
**DBIL**	Direct bilirubin	Liver-related	μ mol/L	1.7-6.8 μ mol/L	183	3.42	1.14
**GGT**	Gamma-glutamyl transferase	Liver-related	U/L	10-47 U/L	183	21.02	14.45
**TBIL**	Total bilirubin	Liver-related	μ mol/L	2-20.5 μ mol/L	183	10.94	3.95
**AST/ALT**	AST/ALT ratio	Liver-related		1-3	183	1.35	0.33
**IBIL**	Indirect bilirubin	Liver-related	μ mol/L	1.7-8.3 μ mol/L	183	7.52	2.94
**A/G**	Albumin/Globulin ratio	Protein		1.2-2.4	183	1.70	0.18

**ALB**	Albumin	Protein	g/L	38-51 g/L	183	46.92	1.99
**GLB**	Globulin	Protein	g/L	20-40 g/L	183	27.81	2.65
**TP**	Total protein	Protein	g/L	20-80 g/L	183	74.73	3.13

Figure S1. **Demographic information of recruited participants**

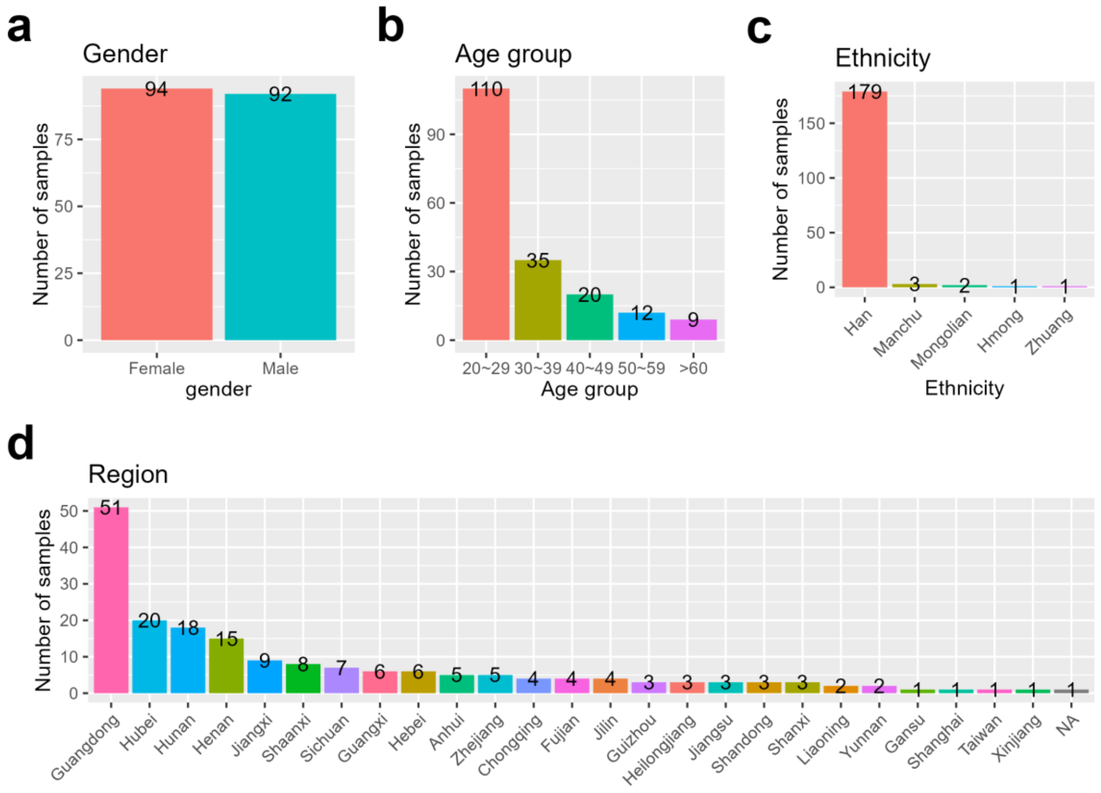

Notes: (a) Gender distribution of the 186 participants; (b) age distribution of the 186 participants; (c) ethnicity information of the 186 participants; and (d) birth origin information of the 186 participants.

Figure S2. Sequencing depth of raw and down-sampled FASTQ files

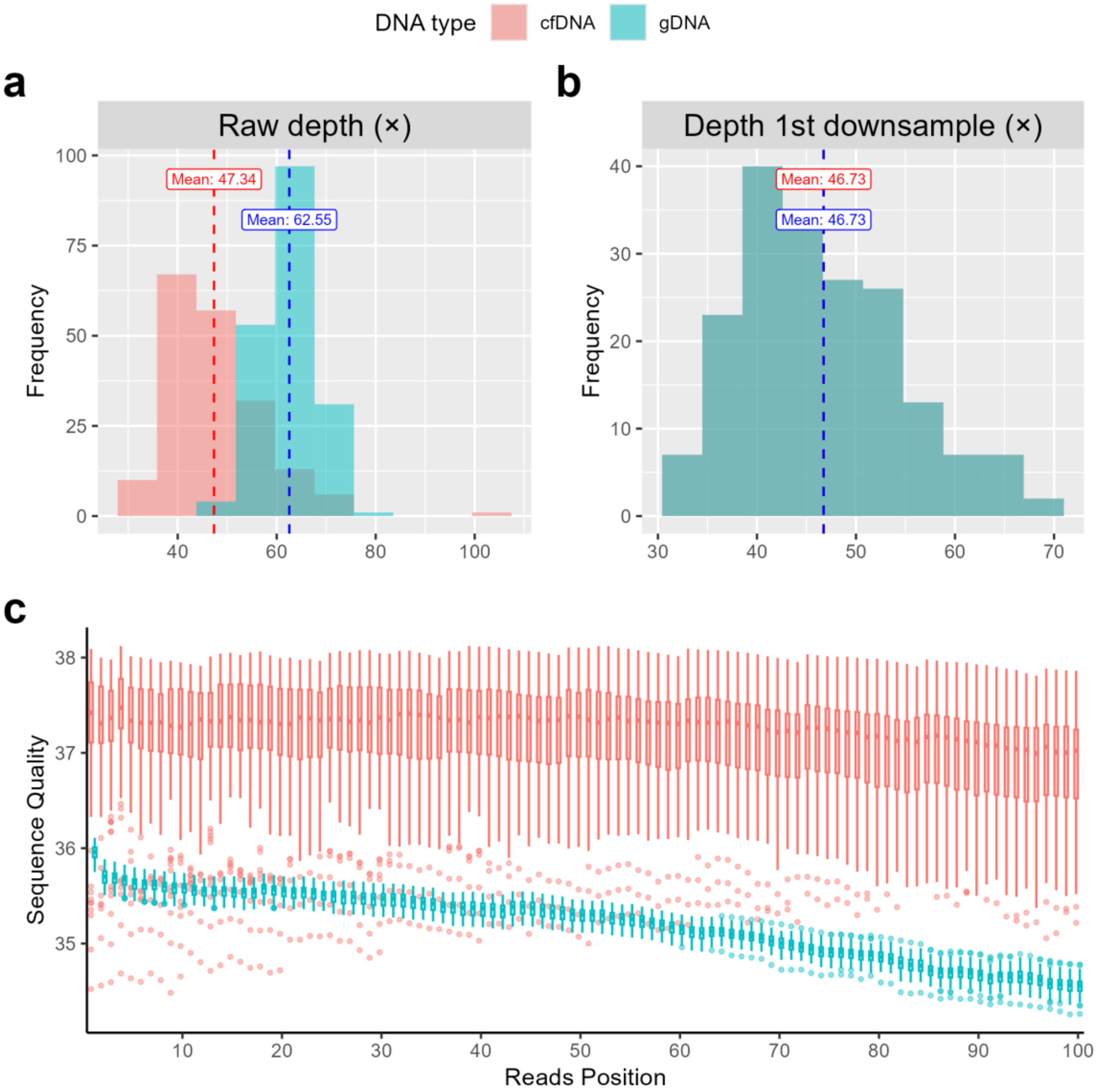

Notes: (a) Distribution of raw sequencing depth for 186 participants in cfDNA and gDNA; (b) distribution of sequencing depth after initial down-sampling in cfDNA and gDNA; and (c) sequencing quality scores for cfDNA and gDNA.

Figure S3. **Depth following read alignment and secondary down-sampling**

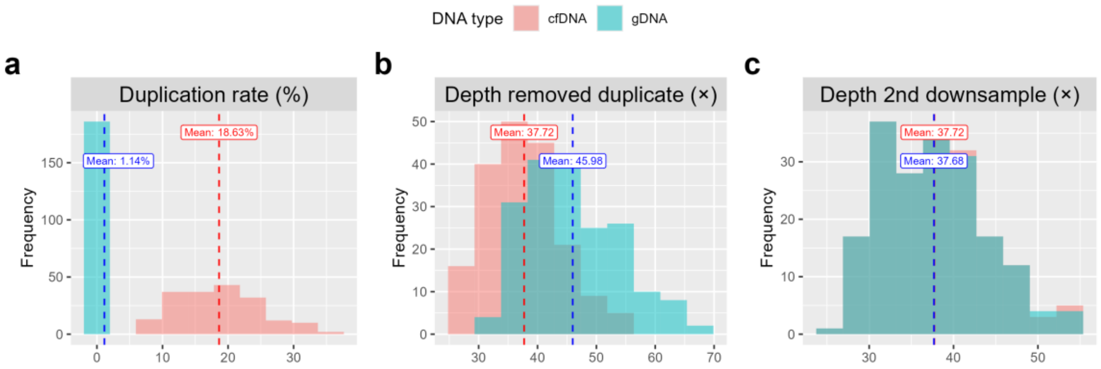

Notes: (a) Distribution of duplicate read rates among 186 participants for cfDNA and gDNA; (b) distribution of sequencing depth after duplicate read removal in cfDNA and gDNA; and (c) distribution of sequencing depth after secondary down-sampling in gDNA.

Figure S4. **Post-alignment metrics**

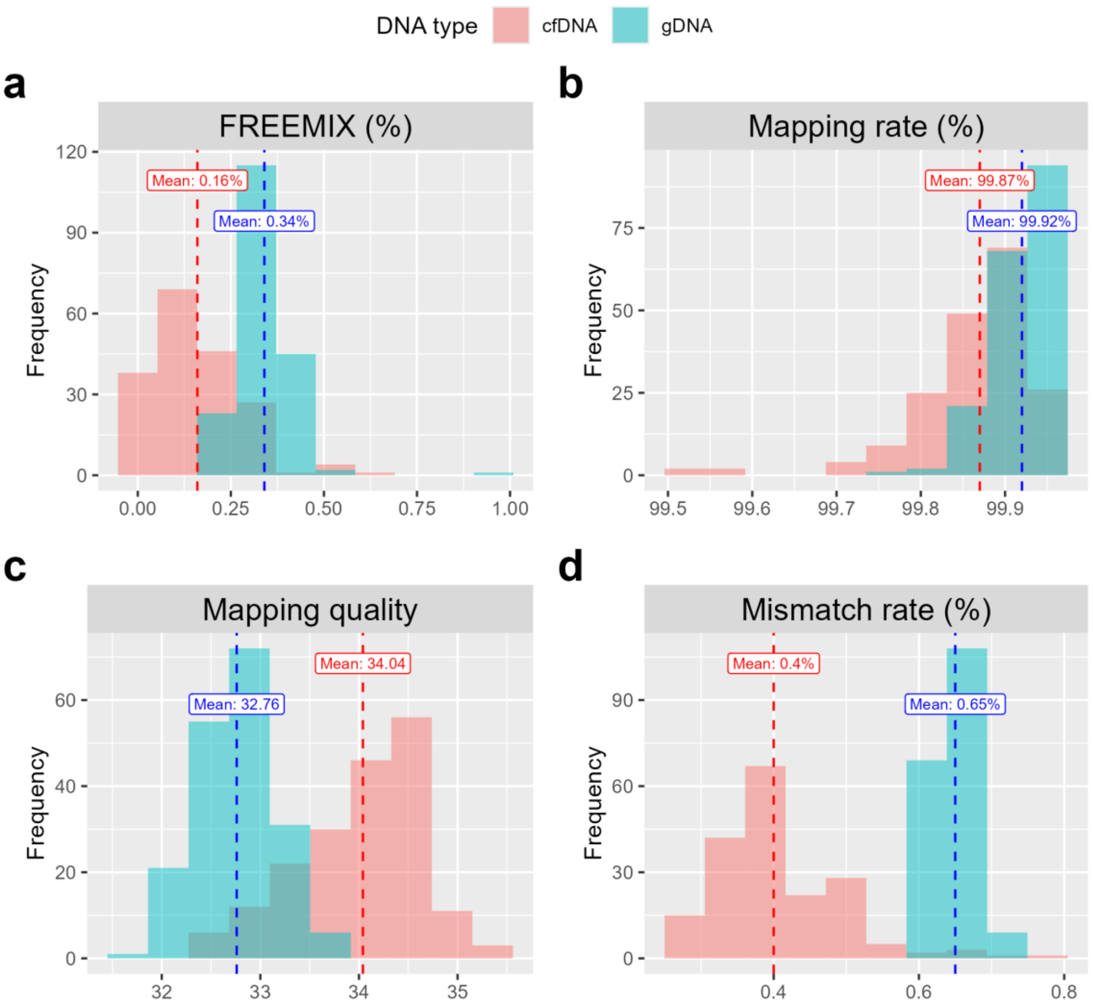

Notes: (a) Distribution of contamination rates (measured by FREEMIX) in cfDNA and gDNA; (b) distribution of mapping rates in cfDNA and gDNA; (c) distribution of mapping quality in cfDNA and gDNA; and (d) distribution of mismatch rates in cfDNA and gDNA.

Figure S5. Base depth distribution of cfDNA and gDNA across 22 chromosomes

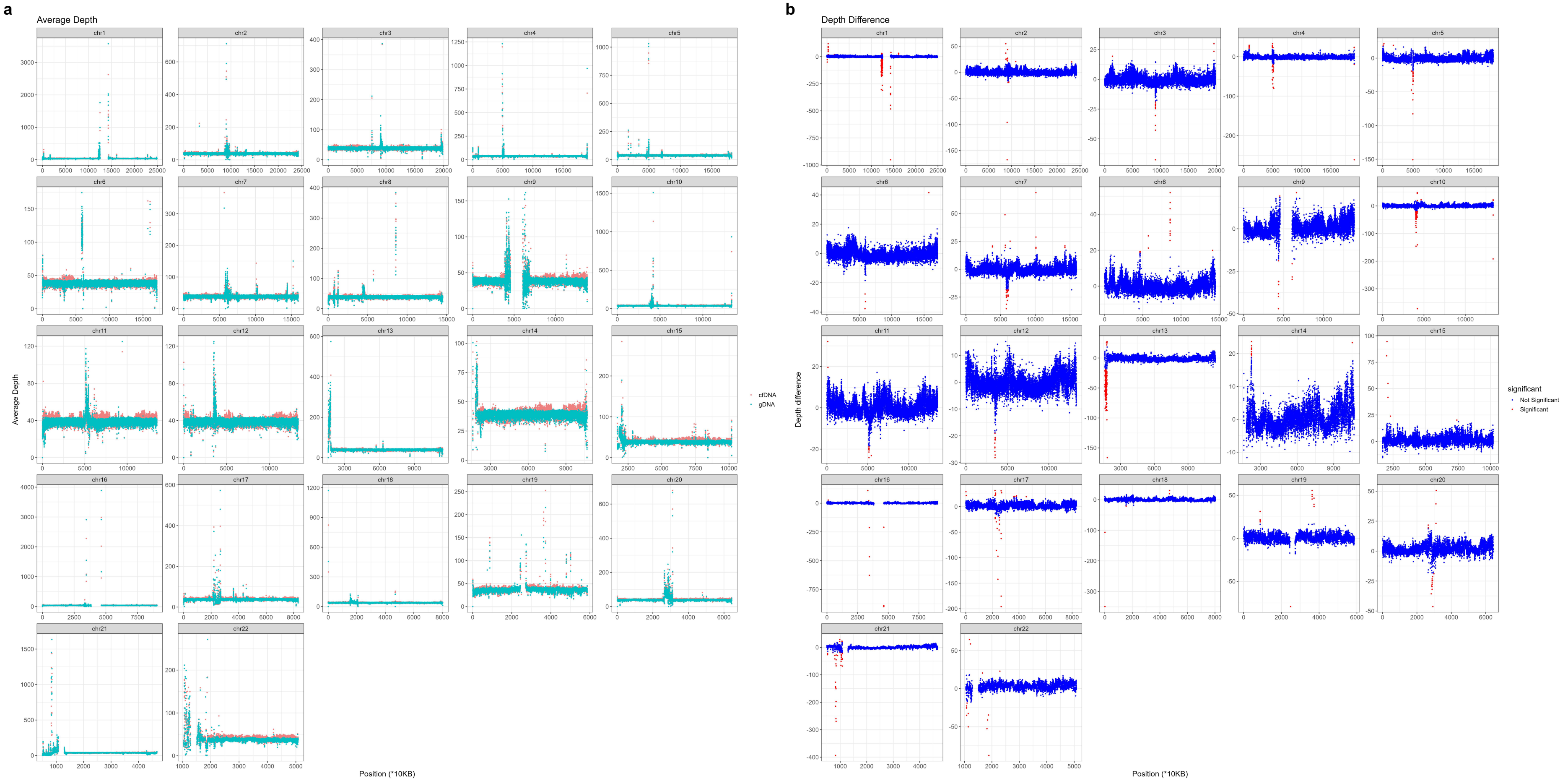

Notes: (a) Depth distribution of bases in cfDNA and gDNA; and (b) differences in base depths between cfDNA and gDNA.

Figure S6. **Venn diagrams of annotations for bases with significant depth differences**

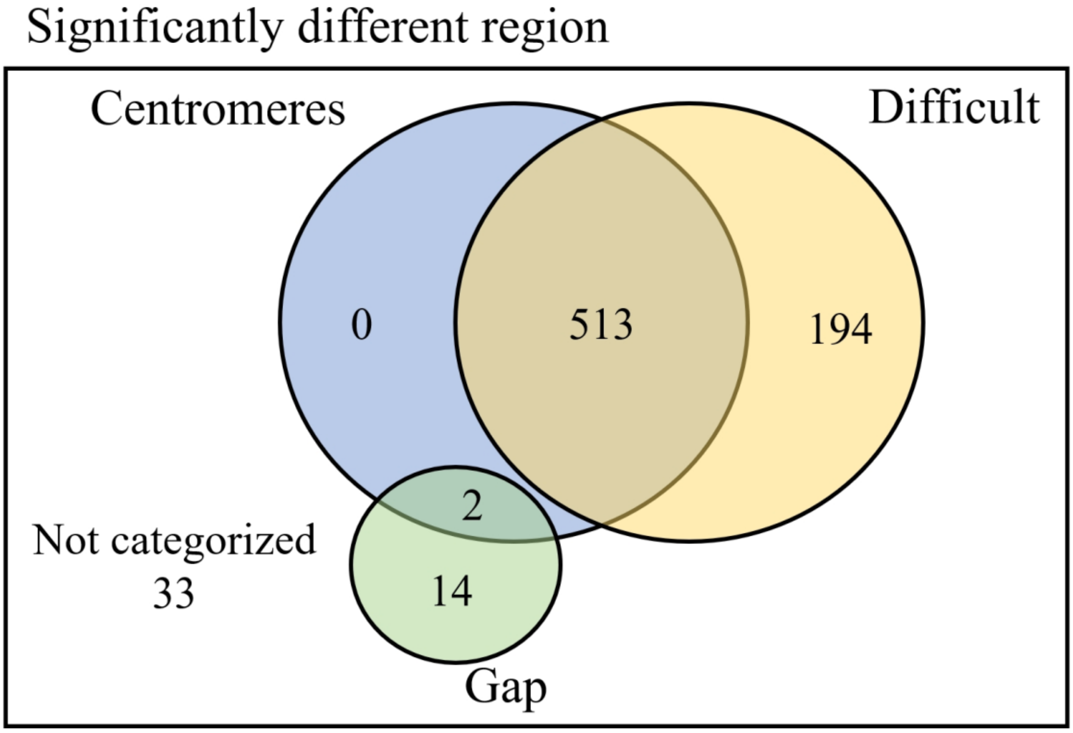

Figure S7. Illustration of coverage distribution of cfDNA and gDNA

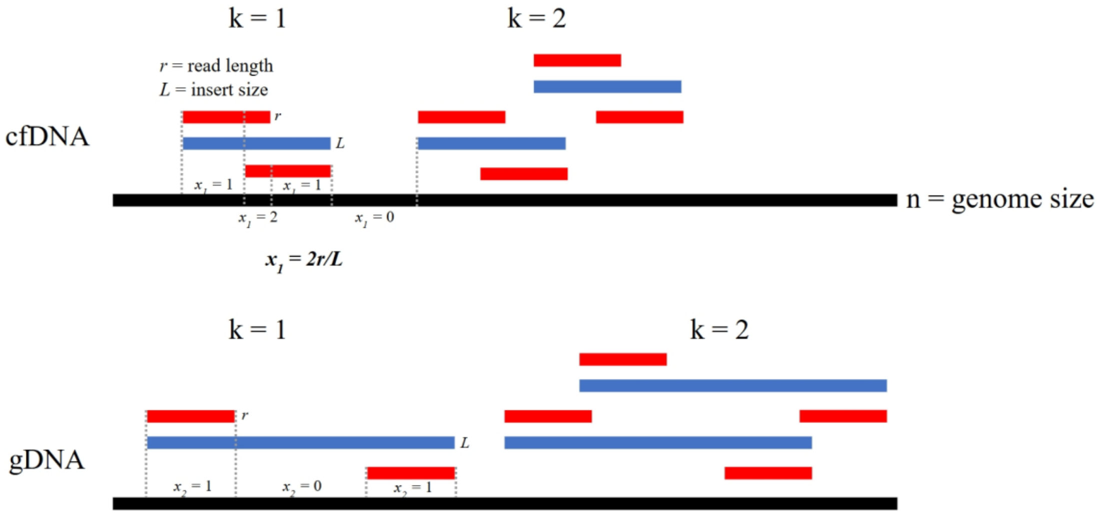

Notes: This figure illustrates the characteristics of two sequencing materials during an actual sequencing process. cfDNA exhibits shorter insert sizes, resulting in variable sequencing depths across different positions of a single DNA fragment. In contrast, gDNA has longer insert sizes.

Figure S8. **Information of genetic variants**

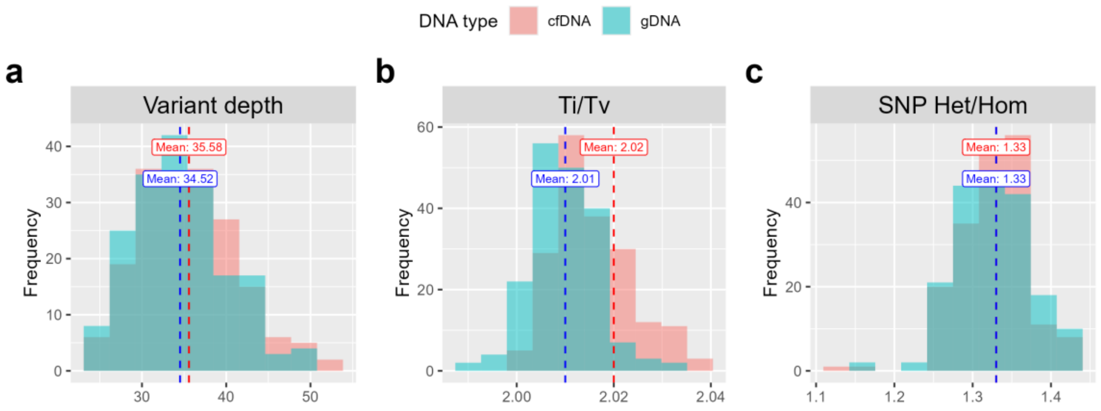

Notes: (a) Distribution of single-nucleotide polymorphism (SNP)-level depth in cfDNA and gDNA; (b) distribution of SNP-level transition/transversion (Ti/Tv) ratios in cfDNA and gDNA; and (c) distribution of SNP-level heterozygous/homozygous (Het/Hom) ratios in cfDNA and gDNA.

Figure S9. Comparison of GWAS performance between cfDNA and gDNA

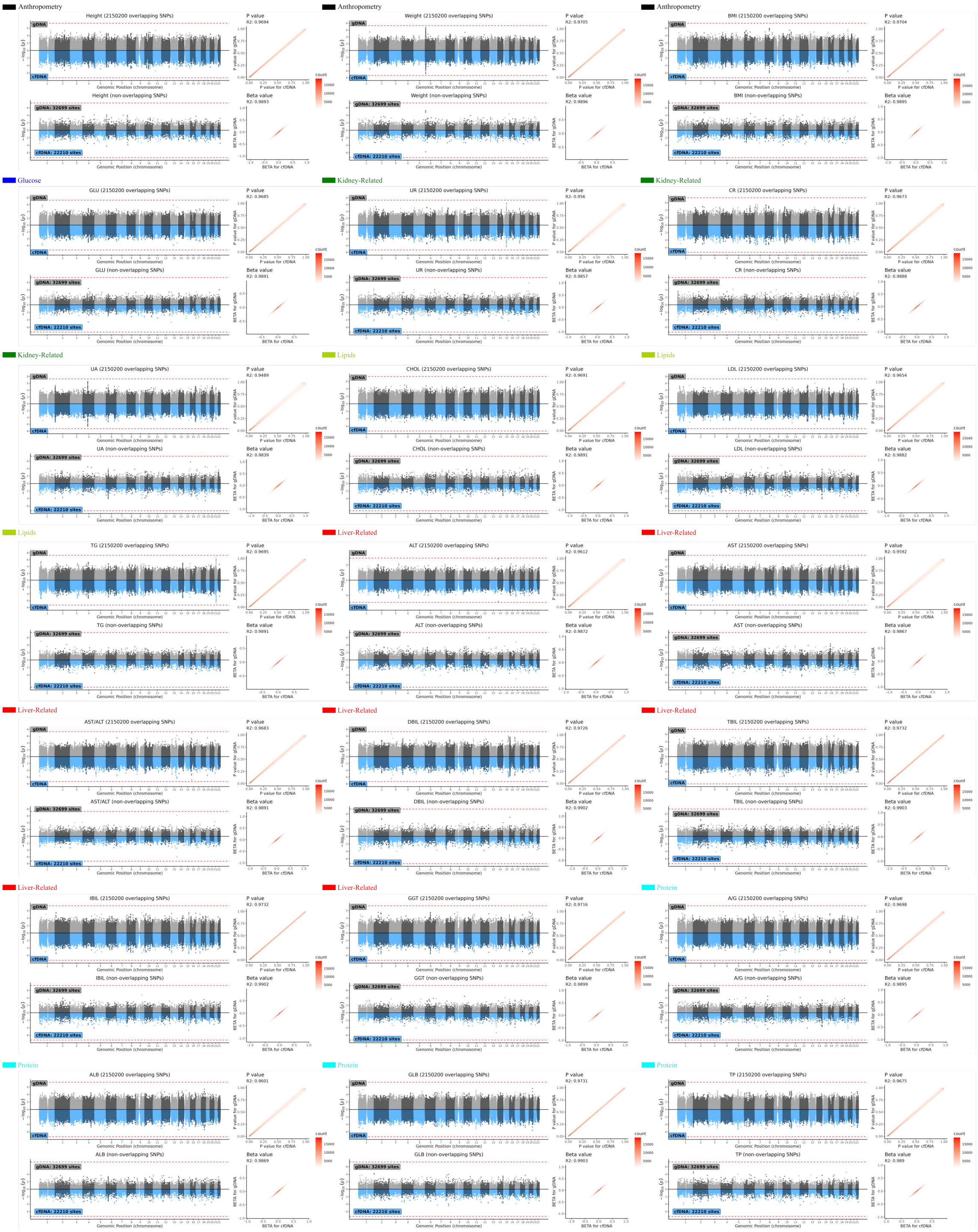

Notes: Mirrored Manhattan plots and scatter plots of p-values and beta values from GWAS results based on cfDNA and gDNA. The phenotypes were grouped into six categories: Anthropometry (height, weight, BMI: body mass index), Glucose (GLU: glucose), Kidney-Related (UR: urea, CR: creatinine, UA: uric acid), Lipids (CHOL: total cholesterol, LDL: low-density lipoprotein cholesterol, TG: triglycerides), Liver-Related (ALT: alanine transaminase, AST: aspartate transaminase, AST/ALT:

AST/ALT ratio, DBIL: direct bilirubin, TBIL: total bilirubin, IBIL: indirect bilirubin, GGT: gamma-glutamyl transferase), and Protein (A/G: albumin/globulin ratio, ALB: albumin, GLB: globulin, TP: total protein).

Figure S10. **Comparison of eQTL performance between cfDNA and gDNA**

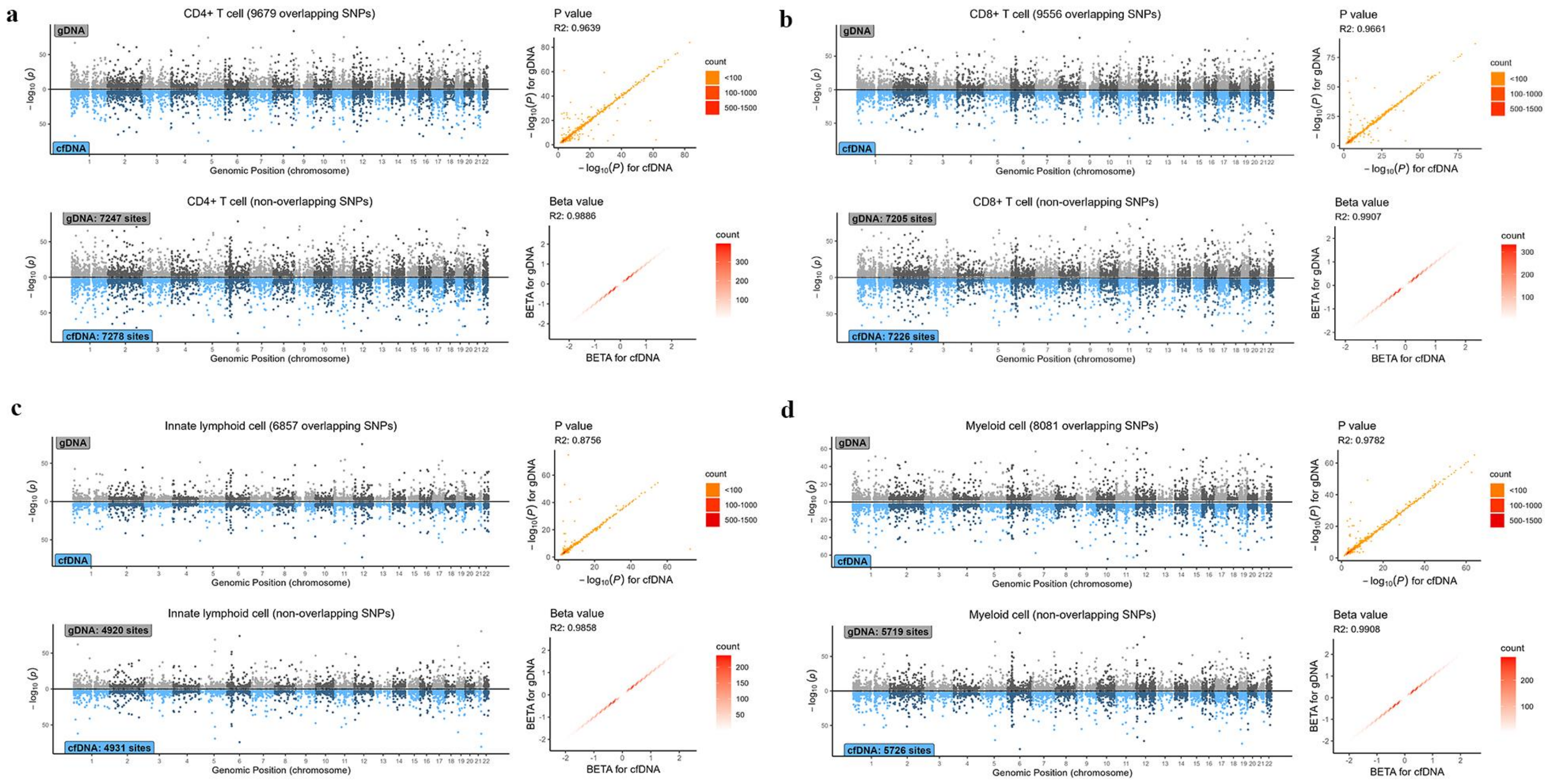

Notes: Mirrored Manhattan plots and scatter plots of p-values and beta values from eQTL analysis results based on cfDNA and gDNA. (a–d) The cell subpopulations shown are CD4+ T cells, CD8+ T cells, innate lymphoid cells, and myeloid cells, respectively.
